# Protocol to develop strategies to improve the effectiveness and efficiency of Village Health Workers in service delivery in a selected district of Zimbabwe

**DOI:** 10.1016/j.mex.2024.102850

**Published:** 2024-07-05

**Authors:** Ofhani Munyai, Azwinndini G. Mudau, Ntsieni S. Mashau

**Affiliations:** Department of Public Health, Faculty of Health Sciences, University of Venda, Thohoyandou, South Africa

**Keywords:** Development, Primary health care, Strategy, Village Health Workers, Research protocol

## Abstract

Village Health Workers (VHWs) in Zimbabwe complement the healthcare staff in primary health care delivery. In 2015 the Ministry of Health streamlined services offered by the VHWs with the VHW Strengthening Plan to improve the effectiveness of the program. However, these continue to offer services not addressing the current and emerging health problems. This three-phased study seeks to develop strategies to improve the effectiveness and efficiency of VHWs in service delivery. Systematic literature review shall be used to develop a conceptual framework to guide the development of VHWs service delivery strategies. Exploratory sequential mixed methods design shall explore VHWs roles in primary health care. A survey in the first stage shall collect qualitative data from 45 purposely selected healthcare workers and VHWs using interviews and then thematically analyzed with MAXQDA. The variables generated will have a cross-sectional survey used to collect quantitative data from 134 VHWs and analyzed on SPSS. The SWOT and basic logic models shall be used to develop strategies validated by the Delphi Technique and Key Stakeholders. Informed consent will be maintained in the study with findings published in journals and presentation symposiums. This protocol was approved by the University of Venda Research Ethics Committee (Registration FHS/23/pH/11/0709).

## Graphical abstract

**Figure:**
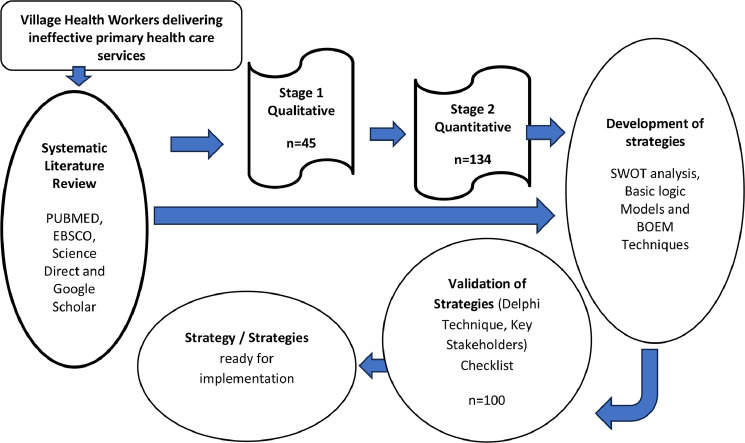

Specifications table

**Table:** 

Subject area:	Medicine and Dentistry
More specific subject area:	Primary health care
Name of your protocol:	Strategies to improve the effectiveness and efficiency of Village Health Workers in service delivery in a selected district of Zimbabwe
Reagents/tools:	N/A
Experimental design:	N/A
Trial registration:	N/A
Ethics:	This study would be conducted in accordance with the principles of the Helsinki Declaration. It was approved by the University of Venda Research Ethics Committee (Registration FHS/23/pH/11/0709) and was approved by the Medical Research Council of Zimbabwe (MRCZ A/3175). Permission will also be sought from all the participating health facilities where all health care workers will be enrolled. Written informed consent would be obtained from all the study participants.
Value of the Protocol:	• The protocol describes in detail reproducible methods to conduct systematic literature review to identify existing strategies to improve service delivery by VHWs. • Development of a conceptual framework as guided by literature on strategies to enhance service delivery by VHWs • Develop and validate strategies that leverage on empirical evidence to enhance the effectiveness and efficiency of VHWs in the delivery of primary health care.

## Background

Countries are working towards the vision of achieving *healthy lives and promoting well-being for all ages*. The delivery of essential health care services through primary health care (PHC) is vital for the realization of universal health coverage (UHC). However, this is hindered by the unsustainable shortages, imbalances and performance of the health workforce [1]. The World Health Organization (WHO) Human Resources Observer Series 19 stated that in 2013 Africa and Asian countries had a combined health worker shortage of 4.2 million [2]. Zimbabwe reportedly had 8 core health workers per 10,000 population in 2015 against the WHO's recommended 23 per 10,000 population [3]. The ‘brain drain’, HIV / AIDS and migration were often cited as the major contributory factors for health care worker shortages [4].

The Global Strategy on Human Resources for Health (2030) affirmed that health care worker shortage compels countries to harness the potential of the Community Health Workers (CHW) in their PHC systems [5]. These have been defined as health care workers who are selected and live in the communities they serve, receive standardized training outside the formal nursing and medical curricula and perform essential health services [6]. The term CHW has been used as an umbrella term across different settings and program contexts such as ‘lady health workers’ (Pakistan), rural health motivator (Swaziland), village health workers (VHWs) in Zimbabwe and health promoters in other countries [7]).

The conference of Alma Ata in 1978 provided a radical declaration towards the achievement of UHC through PHC services which are community driven, equitable and quality health care [8]. It has been shown that VHWs can enable health systems to achieve significant progress in terms of improved effectiveness and efficiency of PHC services through increasing access to preventive and promotive services; early diagnosis and treatment of myriad conditions [9].

The VHW program in Zimbabwe was found to be ineffective in supporting the rural health centers and clinics for promotive, preventive, curative and rehabilitative services at household level under the context of PHC [3]. Poor outcomes have characterized the health system with the infant mortality rate (IMR) having increased from 53 per 1000 in 1992 to 56 per 1000 live births in 2016; and the maternal mortality rate (MMR) remains high at 443 per 100 000 which is a way too high in relation to the SDG target of 70 per 100 000 [10]. Matabeleland South Province has the highest HIV prevalence of 21 % (against the overall 14 % in the Country) of which Beitbridge district contributes 75 % of this proportion [11]. The situation analysis of the community health system recommended for the program to be redesigned to improve its effectiveness, efficiency and sustainability in order for it to continue to play supportive roles of promotive, preventive, minor curative and basic rehabilitative services equitably [12].

The health system in Zimbabwe continues to be overstretched by the chronic shortage of health care workers due to their migration to European countries. Much reliance has now placed on the services of Village Health Workers (VHWs) to bridge this gap through task shifting. However, these continue to face unprecedent challenges in service delivery despite the government putting plans to enhance their effectiveness and efficiency in primary health care. The role and challenges VHWs were explored by Gore et al. [3] while Kambarami et al. [13] studied the factors influencing the performance of these cadres. There has been a dearth of studies in Zimbabwe that developed and validated strategies to improve primary health care service delivery. This study aims to provide a conceptual framework to guide the study in the development and validation of strategies leveraging on empirical evidence and informed by setting and context in order to enhance the effectiveness and efficiency of the VHWs in the delivery of primary health care services.

## Description of protocol

### Rationale of the study

The Government of Zimbabwe has called for the revitalization of the VHW programmes to bridge the gap between the formal health system and the communities to improve on the PHC delivery [13]. Whilst the VHW Strengthening Plan and the Community Systems Strengthening Framework for Health were developed, these were never operationalized [12]. It was reported that in Beitbridge district limited coordination and duplication of community health services by the VHWs on certain population groups with the others remaining underserved [14]. There has been a dearth of studies in Zimbabwe that explored the roles of VHWs with the aim of developing and validating strategies to improve PHC service delivery. This calls for new strategies informed by setting and context to improve on the responsiveness of the system in addressing community health challenges.

### Significance of the study

An exploration of the role of VHWs could assist the Nursing Directorate in Beitbridge District to identify gaps and / or overlaps to aid in the planning for VHW service delivery programs. The study could assist policy makers in Beitbridge to make informed decisions on the allocation of resources to improve the VHW programs. Strategies developed could help the VHWs to deliver effective and efficient community health services which could ultimately contribute to quality health outcomes. The research project provides an opportunity for the VHWs in Beitbridge district to contribute in policy dialogue which might enhance their ownership of the program.

### Purpose of the study

The purpose of the study is to develop strategies to improve the effectiveness and efficiency of VHWs in the delivery of PHC services in Beitbridge District.

### Objectives of the study

The objectives of the study are divided into three phases and staged: systematic literature review; needs analysis; and development and validation of strategies.

***Phase 1: Systematic Literature Review***

*The review question*: What are the strategies that can be used by village / community health workers for effective and efficient delivery of primary health care services as presented in literature?

• To review literature on the health system strategies for effective and efficient delivery of PHC services by VHWs using the Rodgers Evolutionary concept analysis framework.
• To develop a conceptual framework that could guide the study in the development of strategies to improve the effectiveness of VHWs in delivering PHC services.

***Phase 2: Needs analysis***

This phase has its objectives based on the exploratory sequential mixed methods design and are in two stages.

*Stage 1: Qualitative approach*

• To explore the role of VHWs in the provision of PHC services

*Stage 2: Quantitative approach*

(Will be determined by the findings from thematic analysis of the first stage).

***Phase 3: Development and validation of strategies***

To develop and validate strategies that leverage on empirical evidence to enhance the effectiveness and efficiency of the VHWs in the delivery of PHC services.

### Conceptual framework

For the community health services to be effective and efficient in a certain geographic area, they must be delivered in an integrated manner and in consideration of the social determinants of health while being anchored by the local communities. The triple concept of proximity; locality; and health system integration are key elements of this framework [15]. The local area should be regarded as a spatial and social entity influenced by the inhabitants’ social dynamics. A community health delivery system delivery involving preventive, diagnostic, therapeutic, palliative and supportive care services which are integrated with the wider health system. The proximity of the community health system involves two dimensions of spatial and relational. The former involves primary care services provided as close as possible to the local communities and responsive to the health needs while the latter put more emphasis on equitability and culturally acceptable health service delivery [15].

This study will wholly apply this triple conceptual framework in the following ways: Proximity will entail the technical support provided by the nearest health facilities to the VHWs and in turn effectiveness and efficiency of community health services provided by the VHWs. In locality this will be used to refer to the physical and contextual settings and socio-cultural dynamics of the communities as they might be used to influence strategy development. Health system integration in this study shall be used to explore the opportunities and constraints in community health which could be addressed by the developed strategies to improve the VHWs effectiveness and efficiency in service delivery in line with the national health strategy.

### Definition of terms

**Community Health Worker (CHW)** is an all-encompassing term to mean a health care worker who received standardized training outside the normal medical curricula and has a defined role in community health and the larger health system [6]. In this study a CHW is used synonymously with a Village Health Worker (VHW) to mean a lay community-based health care cadre who is trained by the Ministry of Health and Child Care in Zimbabwe and works within their local communities in the provision of PHC services.

**Effectiveness** is the indicator given by the ratio of the result obtained to the one programmed to achieve [16]. In this study it is used to mean the capacity of the VHW to deliver PHC services with intended health outcomes to the local communities.

**Efficiency** is the condition of maximizing the results of an action in relation to the resources used [16]. In this study it shall be used to mean the extent of VHWs using the available resources in providing PHC services to the communities in the most economical way possible.

**Health System** refers to all organizations and people whose actions have a primary intent of promoting, restoring and maintaining health [17]. In this study a health system is used to mean the delivery of PHC services with the aid of VHWs as a response to the needs of the intended local communities.

**Strategy** has generally been defined as the determination of basic long-term goals and objectives of an enterprise and courses of action including resource allocation to achieve that goal [18]. In this study a strategy is a plan of action designed for the VHWs to achieve sustainable essential PHC services.

**VHWs** is a CHW health care worker who received standardized training outside the normal medical curricula and has a defined role in community health and the larger health system [3]. In this study a VHW is used synonymously with CHW to mean a lay community-based health care cadre who is trained by the Ministry of Health and Child Care in Zimbabwe and works within their local communities in the provision of PHC services.

### Methodology

This study will use an exploratory sequential mixed methods design

#### Study approach

**Fig. 1:**
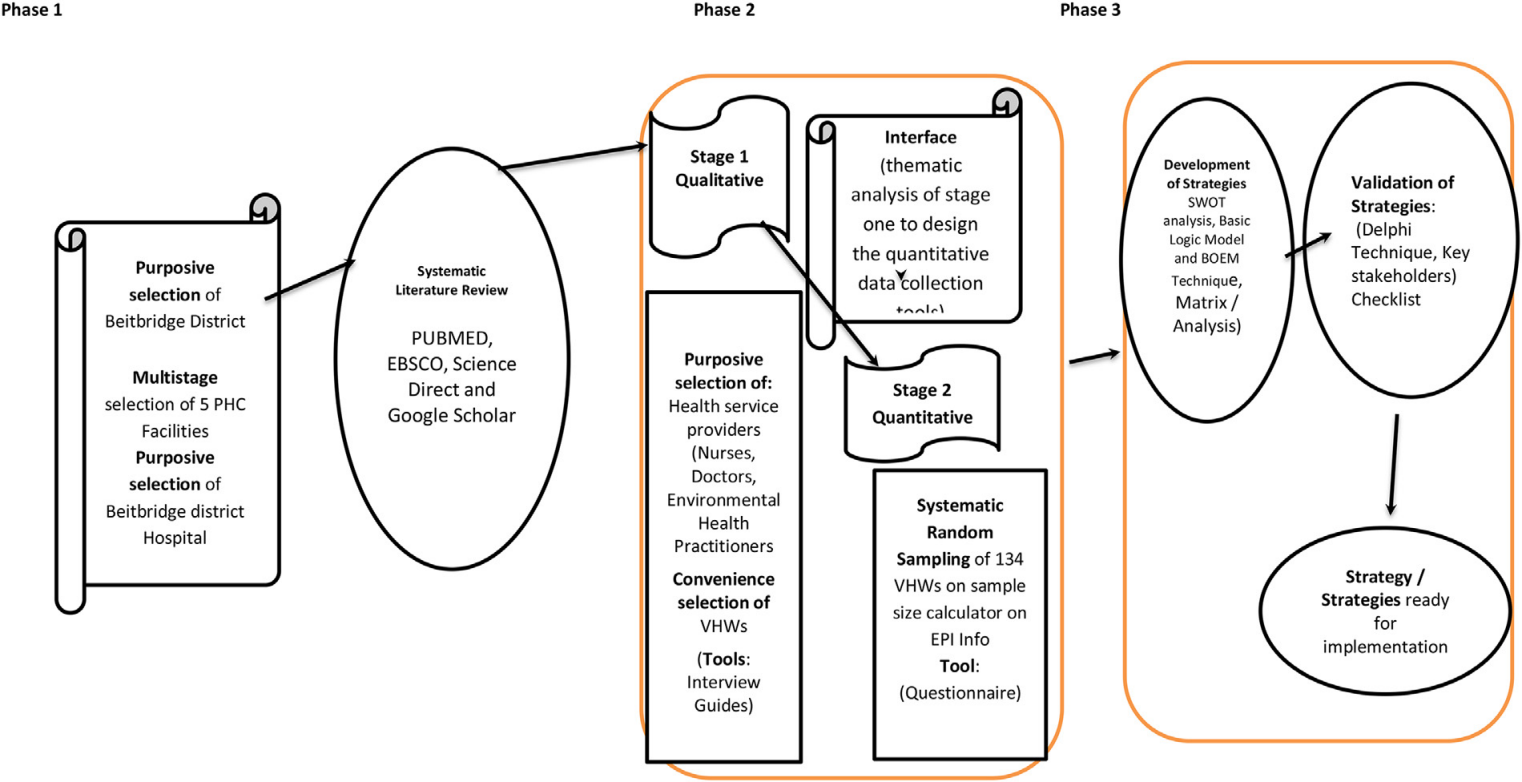
Outline of the research approach and process.

The study will use mixed methodology whereby both qualitative and quantitative approaches will be used. This approach provides for a deeper and comprehensive understanding of the complex problems faced in the social world [19]. This pragmatic worldview places the research question in a central position by using available methods to provide better solutions to the drawbacks of one method by having the strengths of the other complement rather than contending the other. This approach gives the researcher freedom of choice for the methods, techniques and procedures with the focus on the outcomes of the research rather than on the antecedents [19]. Fig. 1 provides an outline of the research approach.

**Phase 1: Systematic literature review**

This phase will utilize the systematic review of relevant literature by the student and the two co-promoters. Systematic reviews use reputable analytical methods to collect secondary data and analyze it and are a type of evidence synthesis formulating research questions through the identification of data directly relating to the systematic review question. They help provide exhaustive summary of literature relevant to the research question [20].

#### Review title

Strategies for effective and efficient delivery of PHC by community or village health workers. A systematic review of literature using the Rodgers Evolutionary concept analysis framework.

#### Review question

What are the strategies for effective and efficient delivery of primary health care services by village / community health workers as presented in literature?

#### Inclusion criteria

The review includes studies that provided PHC service delivery strategies by VHWs / CHWs. Only studies, reports and articles presented in English from January 2010 to December 2022 in peer reviewed journals which include qualitative and / or quantitative research and reports as obtained from PUBMED, EBSCO, ScienceDirect and Google Scholar databases will be reviewed.

#### Exclusion criteria

The review will exclude any studies on other health systems outside the PHC delivery system.

#### Search strategy

Keywords such as: VHW, CHW, PHC, effectiveness and efficiency service delivery strategy, community health system will be used in the search for relevant literature from PUBMED, EBSCO, ScienceDirect and Google Scholar databases.

#### Methods of review

Titles and abstracts to be reviewed by the researcher and checked by the two co-promoters to identify and screen articles that would be relevant to the systematic review. Full texts of articles that meet the inclusion criteria will be further reviewed. Disagreements and differences to be resolved by dialogue until a common ground is found.

#### Data extraction and synthesis

The articles that meet the inclusion criteria will have the Rodger's Evolutionary Conceptual Analysis Framework applied on antecedents and consequences for PHC delivery by VHWs / CHWs. Homogenous data collection criteria shall be determined and agreed upon by the student and co-promoters. Data collected will be compared and any inconsistencies dialogue to reach a consensus between the student and the two supervisors. The findings from the reviewed articles shall be coded and thematically analyzed to identify and explain antecedents, attributes and consequences on the delivery of PHC systems by the VHWs.

### Quality assessments

In line with Rodgers Evolutionary Conceptual Analysis Framework, an evaluation tool for quality assurance shall be used. Further assessments shall be done for clarity on the antecedents, attributes, and consequences of PHC service delivery by the VHWs. An assessment of multiple systematic reviews (AMSTAR) tool for assessing the methodology quality for systematic reviews will also be used [21].

**Phase 2: Mixed methods approach**

A mixed method approach shall be used for this phase where both qualitative and quantitative data will be collected, analyzed, and integrated in one single study. This offers more validity and reliability of the research findings as it has the potential to offset the shortcomings of a single method by balancing with the supremacy of the other [22]. This pragmatic philosophy enables the focus of the study to remain on the social problem to find out what works at the time rather than on methodologies. The mixed method approach will enable the use of the initial phase of the investigation to develop research questions of the latter stage. Through corroboration / confirmation the latter phase will be used to validate the quality of conclusions derived from the initial phase [19].

### Study design

The exploratory sequential mixed methods design [QUAL→QUANT] shall be used whereby qualitative data is first collected and analyzed to explore in depth the viewpoints of the study participants on the role of VHWs in the provision of PHC services to guide the development of research variables for the subsequent quantitative stage. The rationale for using this design is such that the rich qualitative data shall provide an in-depth understanding of the research problem with the identification of important variables for the development of survey instruments. Quantitatively these are tested to enable validation and generalization of the findings to the study population [19]. In this phase, the study shall be conducted in two stages: qualitative followed by quantitative strand (refer to Table 1).

**Table 1:** Outline of exploratory sequential mixed method research process.

Study Phase	Method	Objectives	Participants	Data Collection Method	Data Analysis
Phase 1	Systematic Literature review	• To review literature on the health system strategies for effective and efficient delivery of PHC services by VHWs using the Rodgers Evolutionary concept analysis framework. • To develop a conceptual framework that could guide the study in the development of strategies to improve the effectiveness of VHWs in delivering PHC services.	The review includes studies that provided PHC service delivery strategies by VHWs / CHWs. Only studies, reports and articles presented in English from January 2010 to December 2022 in peer reviewed journals which include qualitative and / or quantitative research and reports as obtained from PUBMED, EBSCO, ScienceDirect and Google Scholar databases will be reviewed.	Titles and abstracts to be reviewed by the researcher and checked by the two co-promoters to identify and screen articles that would be relevant to the systematic review. Full texts of articles that meet the inclusion criteria will be further reviewed. Disagreements and differences to be resolved by dialogue until a common ground is found.	The articles that meet the inclusion criteria will have the Rodger's Evolutionary Conceptual Analysis Framework applied on antecedents and consequences for PHC delivery by VHWs / CHWs. Homogenous data collection criteria shall be determined and agreed upon by the student and co-promoters. Data collected will be compared and any inconsistencies dialogue to reach a consensus between the student and the two supervisors. The findings from the reviewed articles shall be coded and thematically analyzed to identify and explain antecedents, attributes and consequences on the delivery of PHC systems by the VHWs.

Phase 2 (Exploratory sequential Mixed Method)	*(a) Qualitative study*	• To explore the role of VHWs in the provision of PHC services	The health service providers in Beitbridge district would constitute the study population. These are comprised of District Medical Officer (DMO), Registered General Nurses (RGNs), Community Nurses, Environmental Health Practitioners (EHPs) and the VHWs	In-depth Interviews	Thematic Analysis in MAXQDA software
	*(b) Quantitative study*	4. (Will be determined by the findings from thematic analysis of the first stage).	VHWs	Questionnaire with closed questions	Cross Tabulations and Multiple Logistic Regressions in SPSS Version 24 software

Phase 3	*(a) Development of strategies and Validation of developed strategies*	5. To develop and validate strategies that leverage on empirical evidence to enhance the effectiveness and efficiency of the VHWs in the delivery of PHC services.	Analysis of Data from participants in phases 1 and 2 Experts Key Stakeholders	SWOT Matrix Basic Logic Model BOEM Delphi Technique Checklist	SWOT analysis would involve the analysis of the internal and external factors in the VHWs program Expert feedback Quantitative and qualitative analysis of data from the checklist

### Study setting

**Fig. 2:**
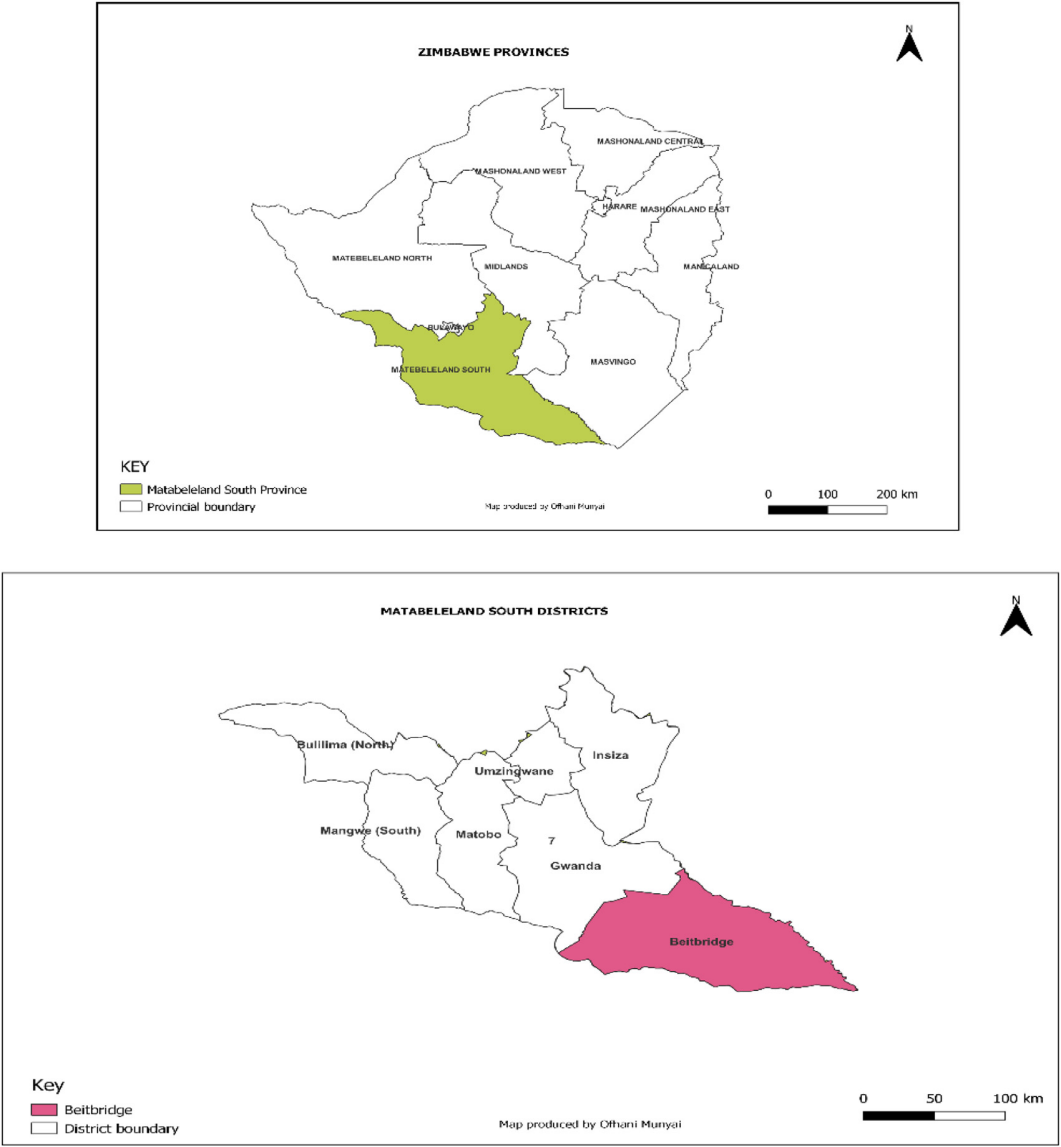
Map of Zimbabwe showing provincial boundaries and Beitbridge district.

The study will be undertaken in Beitbridge district of Matabeleland South Province, Zimbabwe which is the border town with the Republic of South Africa. This district was purposely selected as it is one of the most burdened in the country in terms of maternal, child and reproductive health, and communicable diseases such as TB and HIV which rely on VHWs to provide support on PHC services in terms of community outreach. The district has 15 rural and 6 urban Wards, 20 public healthcare facilities comprising 18 rural health care facilities, 1 clinic and a referral hospital. Most of the people in rural areas rely on VHWs for PHC services due to shortage of professional health care workers in the clinics. The communities speak Venda, Ndebele, Shona and Sotho. It has a population of 152,574 comprising 94,000 rural and 58,574 urban inhabitants. The age distribution is bottom heavy such that over 60 % are between 0 and 35 years which places a high demand in need of PHC services in terms of MCRH. Fig. 2 shows the location of Beitbridge district.

*Phase 2(a): Qualitative Strand*

This phase will use a qualitative assessment for an in-depth exploration of the role of VHWs in the provision of PHC services in Beitbridge district.

### Study population

The health service providers in Beitbridge district would constitute the study population. There are 442 health service providers, and these are District Medical Officer (DMO), Registered General Nurses (RGNs), Community Nurses, Environmental Health Practitioners (EHPs) and the VHWs (refer to Table 2).

**Table 2:** Study population.

Health service provider	Number
Community nurses	8
DMO	1
DNO	1
EHPs	27
RGNs	119
VHWs	286
**TOTAL**	**442**

Source: Beitbridge District Health Information System Two (DHIS2).

### Sampling

Sampling is the process of selecting a small number of study participants from a larger defined target population such that the information gathered from the small group will enable judgments to be made about the larger groups.

#### Sampling of facilities

Since the population is large and hugely dispersed, a multi-stage sampling approach will be used [23]. The first stage will involve division of the study area into district sampling clusters. In this case the district is divided into 3 clusters of East, West and Central. The second sampling stage will involve random selection of 2 PHC facilities from each of the district's 2 rural clusters (East and West) and purposely selecting the urban clinic and the referral hospital. There is one referral hospital and one clinic in the central cluster and these two facilities will be purposely selected. The hospital provides the administrative functions and coordination of the health care system in the district while the urban clinic would be able to determine the scan of PHC services in the urban setting. The total sample of health care facilities from which to draw our study subjects will be 6.

***Phase 2(a). Qualitative strand***

#### Sampling of study participants

The study intends to explore the role of VHWs in the provision of PHC services. Judgmental sampling will be used to select the participants since this information cannot be obtained from other choices. Purposively selected as key informants will be 1 District Medical Officer (DMO), 1 District Nursing Officer (DNO) and 8 Community nurses. These 10 study participants are all stationed at the district referral hospital. The DMO is the chief executive officer accountable for planning, organizing, managing, coordinating and implementing all health-related programs including the VHW activities in the district. The DNO is responsible for assessing district health needs, plans and organizes for implementation in-service training needs for nurses and VHWs, involved in monitoring and evaluation of health programmes. Community nurses work closely with facility-based nurses and health center committees, engage and are also involved in the recruitment of VHWs.

From the selected 5 PHC facilities, 15 health service providers comprising 1 Nurse-in-Charge (NIC), 1 Registered General Nurse (RGN) and 1 Environmental health Practitioner (EHP) from each facility will also be purposely selected as key informants. RGNs and EHPs are more often involved in the support and supervision for the VHWs. In addition to the key informants mentioned above will be 15 VHWs which will be conveniently selected from the 5 selected PHC facilities where each will have 3 members participating. The total sample size is anticipated to be 45.

#### Inclusion criteria

Health service providers comprising curative (DMO, DNO, NICs, RGNs and Community nurses) and preventive (EHPs) staff who are directly involved in support and supervision or directly work with VHWs would be eligible to take part in the study. This target population consists of adult males and females who are above 18 years of age.

#### Exclusion criteria

Health service providers who have not worked in the selected health facilities for at least 6 months prior to data collection will not be eligible for taking part in the study.

### Data collection tool

Semi-structured interview guides, translated in Ndebele, Shona and Venda will be used to collect in-depth qualitative data from the health service providers. These instruments should enable the exploration of the role played by VHWs in the delivery of PHC services. In-depth interview guides enable the researcher to interact with the participants directly to collect data through the elicitation of the feelings, perceptions, viewpoints through probing and helps determine how meanings are shaped with the influence of context [24].

#### Pretest

Pre-test of the in-depth interview guides will be done at two of the non-participating and conveniently selected Chaswingo and Makombe Rural Health Centers in Beitbridge district. A relatively large sample size is required to achieve a reasonably high probability of detecting a problem on the clarity of the questions [25]. The data collection tools will be pretested on 5 (representing 10 % of the sample size) conveniently selected participants comprising 2 nurses and 3 VHWs. This sample size is considered enough to achieve data saturation and therefore test the clarity of questions to enable adjustments to be made on the interview guides [25]. The transcripts will be transcribed verbatim and analyzed to enable adjustments to be made to improve on its construct validity.

### Data collection

Prior arrangements with the responsible authorities from health facilities will be done before visit by the principal researcher and two research assistants. The research team would first administer an informed consent form for participants’ comprehension and signing before the interviews. This would ensure that all the study participants are as comfortable as possible following research ethical principles. In-depth semi-structured interviews will be conducted with key informants: DMO, DNO and community nurses at the District Hospital; NICs, RGNs and EHPs at their respective PHC facilities and VHWs in the venues which are convenient for each one of them and in a language of their preference. The interviews will be tape recorded using a digital tape recorder and transcribed verbatim following consent with each study participant. The in-depth interviews would seek to understand the role of VHWs in the delivery of PHC. Further probing will be done on other cross-cutting issues as well as challenges, opportunities and strategies which can be employed to improve the effectiveness and efficiency of VHWs in delivering PHC services.

### Data management and analysis

It is expected that large amounts of qualitative data to include field notes and audio scripts will be generated from in-depth interviews. Data would need to be collected in an archive to ensure easy accessibility of the data; documentation of analyses done; and retention of data and associated analyses after study completion. Inside the archive, each form and unit of data will be stored in a separate computer file in categorized directory folders and these folders will be labeled by site and appropriate subfolders for individual interviews pseudo-named to ensure confidentiality and anonymity of the study participants. All the data will be placed in one ‘working file’ with the original and complete datasets in separate backup files that would eventually be condensed and transformed into the final report. The related chunks of data will be copied and pasted together and instantaneously categorized.

Qualitative data collected from the semi-structured in-depth interviews will be transcribed verbatim, coded, and analyzed in MAXQDA. A six-step model presented by [26] will be used to guide the process:

1. The first step will involve familiarization with the data set through immersion (repeated listening to the tape-recorded audios and / or reading and studying notes to make brief notes on the insights and analytic ideas related to each data item.
2. The second step to involve coding through the identification of data segments that appear potentially interesting, relevant, or meaningful for the enquiry. These will be coded, collated, and then compiled into relevant data segments for each code.
3. In the third step, initial themes will be generated beginning with identification of shared patterns and meanings across the data set, then compilation of clusters of codes and finally collating all the coded data relevant to each candidate theme.
4. The development and review of themes will be done in stage four. This will be through an assessment of whether the themes make sense through a radical revision and consideration of any relationship between and among themes.
5. The fifth step involves refining, defining, and naming themes through ensuring that these are clearly demarcated, with brief synopsis and with informative names for each theme.
6. The final step [6] will then involve writing up with space integration, dynamic / grounded and visual representation of qualitative data with themes [26].

**Trustworthiness:** The ‘Four-Dimensions Criteria’ put forward by Lincoln and Guba (1985) in will be used as a guiding framework for establishing the trustworthiness of the qualitative data. This will ensure that the research is credible; dependable; confirmable and transferrable in order to guarantee the reliability and validity of the research findings.

**Credibility:** Credibility is to ensure that the study participants concur with the research findings [27]. The researcher will maintain a prolonged engagement with the study participants to have their trust and objectivity to the study findings. The other technique to ensure credibility will be the use of semi-structured interview guides which will be administered to different kinds of participants such as health service providers and VHWs. Data collected from each of the above-mentioned techniques will be triangulated and continuously confirmed with the tape records. The other check would be done by the research supervisors who will provide a quality check on the robustness of the data collection and analysis. It is also envisaged that debriefing will be done to some selected participants in the participating districts.

**Dependability**: To ensure dependability, the researcher should produce a well-researched, logical, traceable and clearly documented study process [28]. This would entail a designed research methodology with robust sampling, data collection and analysis and reporting as an integrated system. The University of Venda panel of experts would provide audit checks to ensure a rigorous research process is followed.

**Confirmability:** Patton in [29] asserted that for the study to be confirmable, the researcher's interpretations on the findings must be informed by the experience and ideas of the informants rather than the enquirer's preferences. The researcher would ensure high levels of objectivity in data collection and analysis. The researcher will declare any presuppositions that they might have. An audit trail will have to be provided for an independent reviewer to trace the research process systematically to determine how data derived from the study led to the eventual recommendations [28].

**Transferability**: Transferability relates to the extent to which the study findings can be transferred to other settings or to a wider population [27]. The researcher will provide a robust contextual description of the settings to enable transferability to other settings by the prospective readers. A sufficiently thick description will be provided to allow readers to compare phenomenal instances in different settings.

***Phase 2(b): Quantitative strand***

This stage will be informed by the findings from thematic analysis of stage one to design the quantitative data collection tools. A cross sectional survey will be used to collect quantitative data with cross tabulations and multiple logistic regression used to analyze the data. This design enables the analyses of data on multiple variables from a population which were investigated at the same point in time to assess how frequently or widely distributed specific variables are in a population [30]. Variables tested will enable generalization of the findings in terms of roles and contextual factors in the VHW program in Beitbridge district.

#### Study population

For the quantitative strand of the sequential exploratory mixed methods design, the target population will consist of VHWs in Beitbridge district.

#### Sampling and sample size

Sampling is a technique of selecting a subset of the population to make statistical inferences about the characteristics of the whole population [31]. The sampling frame will be health facility registers for the VHWs which has 286 VHWs. Calculating using an EPI INFO Software (Version 7), at a Confidence Interval of 95 %; a Margin Error of 5 %; Design Effect of 1; and an Expected Frequency of 50 %, a sample size of 134 VHWs is obtained.

#### Sampling of respondents

Systematic random sampling will be used to select the study respondents with the district register for the VHWs providing the sampling frame. The sampling interval will be 2, having been obtained by dividing the target population, in this case the total number of VHWs (286) in the district by the sample size of 134. The first person on the VHW register will be selected as the first respondent since the sampling interval is 2. Systematic random sampling was chosen because of its simplicity, suitability for use in a hugely dispersed population and results are generally a true representative of the target population [32,33].

#### Inclusion criteria

All the VHWs in Beitbridge district who are in the health facility registers and have worked for at least 6 months in the same health facility will be eligible for selection to participate in the study.

#### Exclusion criteria

VHWs who would have participated in the first phase of the research would not be eligible to take part at this stage.

#### Data collection instrument

A questionnaire consisting of close-ended questions will be used to collect quantitative data. The instrument will be crafted after the analysis of the first phase (qualitative data) for the research as themes will be used to inform the development and testing of variables.

#### Pretesting of data collection instrument

Pretesting of the questionnaire will be done on 13 (representing 10 % of the sample size) randomly selected VHWs at Makombe and Chaswingo Rural Health Centers in Beitbridge district to test on the clarity of the questions. This sample size is expected to achieve a power of 80 % to detect a problem in the clarity of questions [25]. This would help ensure that any unclear questions are rephrased.

**Validity**: Validity refers to the extent at which a data collection instrument measures what it intends to measure and thus the degree to which the results are truthful and has two essential parts: internal (credibility) and external (transferability) [34]. Content validity refers to the extent on which the questionnaire and the scores represent all possible questions that could be asked [34]. To effectively evaluate content validity, a four-step procedure shall be followed: identification and outlining the subject domain of interest; gathering resident domain experts; developing consistency matching methodology; and analysis of the results from matching [34]. Content validity of the data collection questionnaire will be ensured through pre-testing of the tool to enable any adjustments to some of the questions to be made. Face validity will also be measured by reliance on expertise of the assessors on the subject area in order to ascertain measurement of the intended construct under study.

**Reliability:** Reliability refers to the extent to which a measurement of a phenomenon provides stable and consistent results under constant conditions [31]. Consistency across time (test-retest reliability) shall be ensured through retesting the same questionnaire on the same respondents with the scores correlated between two separate measurements to assess external consistency. The inter-rater reliability, being the extent to which information being collected in a consistent manner shall also be obtained through the correlating scores using the Cronbach's Alpha (α) Coefficient for two or more independent raters. After the initial pre-test, the survey questionnaire will be re-administered to the same respondents through a test re-test to measure internal consistency and thus repeatability of the results. Here single items within the questionnaire will be correlated to estimate a coefficient of reliability. (31)maintained that for exploratory or pilot studies, the reliability should be at least 0.60. This would help to improve consistency of the data collected while data cleaning before capture into the analysis software would enable stability of the data and ultimately dependability of the research findings.

### Data collection

A questionnaire with close-ended questions would be administered to the selected VHWs through two trained field assistants and the principal researcher. It is expected that for each respondent it should take between 15 and 20 min to complete a questionnaire. The questions will include demographics and variables derived from the thematic analysis of the qualitative phase.

### Data analysis

IBM Statistical Package for Social Scientists (SPSS) Version 24 will be used to enter data to enable cross tabulations and multiple logistic regressions to be conducted to establish the extent of influence between / among the contextual factors / variables and cross-cutting issues on PHC delivery by VHWs. The results would be presented in tables, pie charts and graphs.

### Phase 3: development and validation of strategies for VHWs service delivery

The development of service delivery improvement strategies and validation would involve the analysis of the findings from the first and second phases of the study. Experts in the field of Health systems and policy and key stakeholders would be enrolled to validate the strategies aimed at enhancing the effectiveness and efficiency of the VHW Program in the delivery of PHC services.

### Strategy development

Strategies development would leverage on literature review and the empirical findings of the sequential exploratory mixed method. Data would be collected based on the SWOT analysis; BOEM and Basic Logic Models. The methodology for strategy development as put forward by [35,36] would also be explored where a series of steps would guide the process starting with the assessment of the relevant internal and external factors that might impact on the delivery of community health services by the VHWs for the long term. Secondly, the indicators would be determined for the evaluation of proposed strategies benchmarked against the empirical findings from literature. The third step would involve development of a correlation regression model of VHW potential to deliver effective and efficient community health services.

**SWOT analysis**: Strength Weakness Opportunity and Threats (SWOT) would involve the analysis of the internal and external factors in the VHWs program. The former are within the control of the organization and includes: financial resources; physical resources (equipment; human resources) while the latter are not under the control of the organization / Ministry such as: market trends; demographics; political and economic environments [37].

**Program Logic Models**: This model will be used to assess the feasibility in terms of implementation of each of the proposed strategies generated from the SWOT analysis. In particular, the appraisal would determine economic sustainability of the inputs; the feasibility of activity implementation and the outputs realized; and the extent of contribution of the possible outcomes to the impacts / goal of the health system as spelt out in the national health strategy. A logic model typically offers a simplified version of the series of steps of a program's operations from inputs to processes/ activities to outputs and then outcomes contributing to the impact [38].

**BOEM Models**: The Build Overcome Explore and Minimize approach will also be used alongside SWOT and Logic Models as the final step in the development of VHWs service delivery improvement strategies. The model, sometimes referred to as a spiral, reflects an underlying concept that each cycle involves a progression that addresses the same sequence of steps. Each spiral commences with the identification of: (a) objectives for the strategy in terms of functionality and ability to accommodate change; (b) the alternative means of implementing the strategy; and (c) the constraints imposed on the application of the alternatives in terms of costs, schedule, and interface. The following step will entail the assessment of each alternative strategy relative to the objective and constraints. This process will help identify areas of uncertainty that could be sources of strategy risk. The next step would involve formulation of cost-effective strategy for resolving potential sources of risks. The BOEM model basically involves: prototyping; simulation; benchmarking; reference checking; administering user questionnaires, analytic modeling or other risk resolution techniques [39].

### Strategy validation

Strategy validation aims at determining feasibility, applicability, acceptability and sustainability of strategies in order to realize the intended outcomes [36]. Two methods will be employed: empirically using the Delphi Technique; and then use of stakeholders from the district under study.

**Delphi technique**: The Delphi technique will constitute one of the two methods that will be used to validate the VHW service delivery improvement strategies. The Delphi technique is a structured, systematic, interactive, and forecasting technique of communication which relies on a panel of subject area experts who answer questionnaires in two or more rounds. It has also been described as a qualitative data analysis and consensus reaching technique which provides insights of subject area experts for effective decision making [40]. The panel of experts will be recruited purposely based on their extensive knowledge on health systems and policy based on scholarly publications and / or experience in the field. Following engagements for their consent these will be appraised of the research findings from the prior phases and then requested to critique the proposed VHW service delivery improvement strategies. A statistical summary will be produced from the responses with the process continuing until a level of stability is reached.

**Stakeholder validation:** A statistically significant and representative sample of stakeholders from the Ministry of Health and Child Care, VHWs and local communities will be selected from Beitbridge district for the purpose of strategy validation. A data collection questionnaire will be administered to measure the feasibility of implementation, applicability, and sustainability of each of the proposed strategies. The results will then be analyzed statistically using SPSS software.

### Ethical considerations

Outlined below are the ethical considerations for this study

#### Ethical clearance

The study proposal and data collection tools were subjected to quality assessments by the Department of Public Health and Faculty of Health Sciences. This was followed by submission to the University of Venda Research Ethics Committee for clearance (FHS/23/pH/11/0709) and was approved by the Medical Research Council of Zimbabwe (MRCZ A/3175). Permission will also be sought from all the participating health facilities where all health care workers will be enrolled.

#### Informed consent

The researcher will ensure that the study participants are well-versed with what will be asked of them, how the data will be used and possible consequences (if any) that might arise through the provision of information leaflet which will be attached to the consent form. They will be made to understand their rights in the study which also include access to the information provided and / or to withdraw from participation at any point in time. A written informed consent form will be used as a contract between the researcher and the study participants. Informed Consent entrenched in the Nuremberg Code, the Declaration of Helsinki and the Belmont Report, is a legal requirement for any research involving human subjects [41]. This principle aims to ensure that participants are informed about all the aspects of the research for them to voluntarily confirm their willingness to take part [41].

#### Anonymity and confidentiality

The researcher would guarantee that the identity of the study participants is kept anonymous and confidential by ensuring that the data collection instruments do not include self-identifying statements. Anonymity and confidentiality are important for the protection of the study participants from potential harm such as: reputational; physical; emotional; and / or resource loss [42]. The research design would ensure avoidance of harm to the individual, community and institutional. In instances where the identity of the study participant is known to the researcher, such data shall be de-identified. Data will be kept safe by locking it in a cupboard and the tapes will be destroyed upon completion of the study.

#### Principle of beneficence

The researcher would try by all means to safeguard the interest of the study participants. [43] emphasized the importance of study designs to ensure minimization of the risk to the participants and maximize the benefits to the society. The participants would also be asked to identify any possible risks in their participation in the study to help mitigate against harm. The in-depth interviews will be conducted in a non-intimidating environment and should any participant show signs of distress, they will be counseled.

#### Principle of fairness and justice

The researcher would ensure appropriate treatment of study participants and that distributive justice is followed throughout the study from data collection to validation of the strategies. Equal chance shall be afforded to all participants. The principle of justice provides for fair procedures and impartiality by the research team in order to make unbiased decisions. [43] stated that the principles of justice and fairness are central to the procedure that generates unbiased, consistent and reliable decisions and enables acceptance of the study findings.

### Plan for dissemination and implementation of results

The process of validation would ensure preliminary dissemination of the strategies and for the participants to realize their contribution in the research as this would also form part of de-briefing. The researcher would publish the research findings in scholarly journals in the form of papers and presented in research symposiums.

## CRediT authorship contribution statement

**Ofhani Munyai:** Conceptualization, Methodology, Data curation, Writing – original draft. **Azwinndini G. Mudau:** Supervision, Writing – review & editing, Validation. **Ntsieni S. Mashau:** Supervision, Writing – review & editing, Validation.

## Declaration of competing interest

The authors declare that they have no known competing financial interests or personal relationships that could have appeared to influence the work reported in this paper.

## Data Availability

No data was used for the research described in the article.
